# Long-term Dynamics of Measles Virus–Specific Neutralizing Antibodies in Children Vaccinated Before 12 Months of Age

**DOI:** 10.1093/cid/ciae537

**Published:** 2024-11-04

**Authors:** Maaike van der Staak, Hinke I ten Hulscher, Alina M Nicolaie, Gaby P Smits, Rik L de Swart, Jelle de Wit, Nynke Y Rots, Robert S van Binnendijk

**Affiliations:** Center for Infectious Disease Control, National Institute for Public Health and the Environment, Bilthoven, The Netherlands; Department of Viroscience, Erasmus MC, Rotterdam, The Netherlands; Center for Infectious Disease Control, National Institute for Public Health and the Environment, Bilthoven, The Netherlands; Center for Infectious Disease Control, National Institute for Public Health and the Environment, Bilthoven, The Netherlands; Center for Infectious Disease Control, National Institute for Public Health and the Environment, Bilthoven, The Netherlands; Department of Viroscience, Erasmus MC, Rotterdam, The Netherlands; Center for Infectious Disease Control, National Institute for Public Health and the Environment, Bilthoven, The Netherlands; Center for Infectious Disease Control, National Institute for Public Health and the Environment, Bilthoven, The Netherlands; Center for Infectious Disease Control, National Institute for Public Health and the Environment, Bilthoven, The Netherlands

**Keywords:** measles, MMR vaccine, immunity, early infant vaccination, long-term immune protection

## Abstract

**Background:**

Measles is a highly contagious disease, presenting a significant risk for unvaccinated infants and adults. Measles vaccination under the age of 12 months provides early protection but has also been associated with blunting of antibody responses to subsequent measles vaccinations and assumed to have lower vaccine effectiveness.

**Methods:**

Our study included children who received an early measles, mumps, and rubella (MMR) vaccination between 6 and 12 months of age (n = 79, given in addition to the regular MMR vaccination schedule at 14 months and 9 years) and a group without additional early vaccination (n = 44). We evaluated measles virus (MeV)–specific neutralizing antibodies before vaccination at 14 months and up to 6 years thereafter using a plaque reduction neutralization test according to the standard set by the World Health Organization.

**Results:**

We found a significant association between age of first MMR and MeV-specific neutralizing antibody levels later in life. Although most children who received early vaccination seroconverted after the first dose, children vaccinated before 8.5 months of age exhibited a markedly faster antibody decay and lost their protective neutralizing antibody levels over 6 years.

**Conclusions:**

Routine vaccination of infants under 8.5 months of age may lead to blunted MeV-specific antibody responses to subsequent MMR vaccination. Early MMR vaccination should only be considered during measles outbreaks or in other situations of increased risk of MeV infection.

**Clinical Trials Registration**. EudraCT 2013-003078-28.

## Teaser

Children vaccinated before 8.5 months of age lose their protective neutralizing antibody levels within 6 years. Early measles, mumps, rubella (MMR) vaccination should only be considered during measles outbreaks or in other situations of increased risk of measles virus infection.

## Graphical Abstract

**Graphical Abstract:**
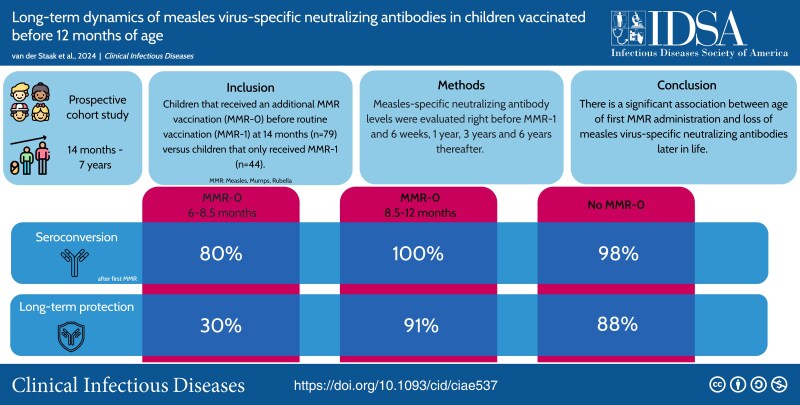
This graphical abstract is also available at Tidbit: https://tidbitapp.io/institutional-portal/clinical-infectious-diseases/tidbits/long-term-dynamics-of-measles-virus-specific-neutralizing-antibodies-in-children-vaccinated-before-12-months-of-age/update

Measles is a vaccine-preventable viral disease characterized by fever and skin rash [1]. Measles virus (MeV) is highly contagious and transmitted via the respiratory route. Infants stand out as the most vulnerable if infected, facing an increased risk of measles-related complications such as bronchopneumonia and acute diarrhea (87.5% for children <1 year of age compared to 64.7% for those >1 year of age) or subacute sclerosing panencephalitis (1:609 for children <1 year of age compared to 1:1367 for children <5 years of age) [2, 3]. In the first months of life, infants are protected from infection by maternal antibodies. The gradual decay of these antibodies is influenced by various factors, such as the immune status of the mother and duration of pregnancy [4].

Vaccinated mothers have, on average, lower MeV-specific antibody levels than mothers who experienced measles during childhood [5]. Consequently, they transfer lower levels of maternal antibodies to their infants, who become susceptible to MeV infection at an earlier age. In recent years, this has resulted in calls to expedite first MeV vaccinations. This becomes even more urgent in outbreak settings, when unvaccinated infants are at imminent risk of exposure to MeV [6]. In these cases, the World Health Organization (WHO) recommends giving the first measles-containing vaccine (MCV1) as early as 6 months [7, 8]. In regions marked by high measles incidence and mortality in infancy, MCV1 is programmatically already provided at 9 months of age, which narrows the window of susceptibility [7]. In countries where measles infection occurs later in life, MCV1 is usually administered as of 12 months of age, in particular to avoid interference of maternal antibodies, but also because suboptimal immune responses to vaccination have been described at younger ages [8–11].

Several studies reported that, while vaccination before 9 months of age generally led to similar seroconversion rates, the antibody levels achieved may not be as robust as those seen with vaccination at older ages [11, 12]. Furthermore, it was suggested that vaccine effectiveness improves with age of the first measles vaccination, and the timing of the initial dose significantly influences effectiveness [13, 14].

In response to a major outbreak of measles in the Netherlands in 2013, infants aged between 6 and 12 months, residing in regions where vaccination coverage was below 90%, were offered an additional measles vaccination before routine vaccination at 14 months [15]. We showed that those who received an early measles, mumps, and rubella (MMR) vaccination, especially under 9 months of age, demonstrated a faster decline in MeV-specific neutralizing antibody levels [16]. This study sets out to consolidate these early findings and to explore the long-term impacts of administering the MMR vaccine before 12 months of age by examining MeV-specific neutralizing antibody levels up to 6 years after the MMR vaccination at 14 months.

## METHODS

### Study Details

This study extends a previously described cohort, as documented by Brinkman et al [16], with additional time points up to 6 years post-MMR at 14 months of age (Supplementary Figure 1) [14]. In summary, Dutch infants aged 6–12 months (n = 79) were administered an early MMR-0 vaccination, followed by a second dose at 14 months of age (MMR-1). Additionally, children (n = 42) who only received MMR-1 were included. Exclusion criteria included receiving immunosuppressive medication, having a known or suspected immunological or bleeding disorder, or who were assumed to have had a previous MeV infection, as determined by positive seroconversion before measles vaccination. Because of the latter, 2 children who did not receive early vaccination were excluded. Baseline characteristics of early-vaccinated children and those who were not early-vaccinated were comparable with respect to period of birth, sex, birth year of mothers, duration of pregnancy, and percentage of mothers breastfeeding their children [14]. Serum samples were obtained through finger-prick prior to MMR-1 and 6 weeks (median deviation, −4 days; variation, −14 to 9 days), 1 year (median deviation, −2.1 weeks; variation, −5.3 to 3.7 weeks), and 3 years post–MMR-1 (median deviation, −0.7 weeks; variation, −8.4 to 8.6 weeks). Additional follow-up was done around 6 years (median deviation, 0 months; variation, −55 to 22 weeks) after MMR-1 for both early-vaccinated (n = 48) children and those who were not early vaccinated (n = 17). The study received approval from the Medical Research Ethics Committees United (METC Noord-Holland, Alkmaar, the Netherlands; clinical trial registration: NL45616.094.13).

### MMR Vaccination

Infants received the MMR-VaxPro vaccine (RVG 17672; Sanofi Pasteur–MSD) between 6 and 12 months of age, containing live-attenuated strains of measles virus (Enders’ Edmonston), mumps virus (Jeryl Lynn [level B]), and rubella virus (Wistar RA 27/3). At 14 months of age, this vaccine was administered to all enrolled children.

### Antibody Measurements

The MeV-specific neutralizing antibody titers were assessed using a modified WHO-endorsed plaque reduction neutralization test (PRNT), following a previously established protocol [17, 18]. Seroprotection cutoffs were defined as neutralizing antibody concentrations exceeding 0.12 IU/mL [19].

### Data Analysis

Statistical analyses were performed using RStudio (version 4.3.0; Posit public-benefit corporation). The geometric mean concentration (GMC) of the MeV-specific neutralizing antibody levels was calculated 6 years after MMR-1. For exploratory purposes, the dataset was divided into 1-month age groups based on when individuals received their first MMR vaccination. The intervals and corresponding order levels are as follows: 5.5–6.5, 6.5–7.5, 7.5–8.5, 8.5–9.5, 9.5–10.5, 10.5–12, and 12–15.5 months—the latter representing children who did not receive MMR-0. The GMC and the corresponding 95% CIs of these age groups were calculated by maximum likelihood to account for the presence of observations below the lowest level of quantitation (0.03 IU/mL).

To explore potential age-related variations in MeV-specific neutralizing antibody levels, the data were natural log-transformed and Loess (locally weighted scatterplot smoothing) curves were generated. The asymptotic general independence test, using the coin package (version 1.4-3, Hothorn et al., 2023), was used to assess the association between age at first MMR vaccination and MeV-specific neutralizing antibody levels.

A subset of early-vaccinated children remained below the seroprotection cutoff (0.12 IU/mL) before they received MMR-1 at 14 months of age. The antibody dynamics of these children (n = 7) were compared with children who did not receive early vaccination (n = 17), for which measurements both before MMR-1 and 6 years later were available. The GMC and the 95% CI of these 2 groups were calculated at both time points and the central tendency was compared with the 2-sample Brown-Mood median test using the coin package.

To model the antibody dynamics over time, the dataset was categorized into distinct age groups based on the age at which individuals received their first MMR vaccination. The early-vaccinated children were divided into nearly same-size subgroups, delimited by values that correspond to the first 3 quartiles of the frequency distribution of age, resulting in the following order levels: 5.5–6.85, 6.85–8.48, 8.48–9.88, and 9.88–12 months. Modeling of the antibody dynamics over time was done with a linear mixed-effects model, using the nlme package. The outcome variable was the MeV-specific neutralizing antibody levels before MMR-1 and 6 weeks, 1 year, 3 years, and 6 years later. The fixed-effect structure was constructed as the interaction of the time point and the age group, while the random-effect structure comprised an intercept for the individual and a slope for the time point. The results of the model fit were expressed as predicted means of MeV-specific neutralizing antibody levels with the corresponding 95% CIs and plotted as error bars.

## RESULTS

### Loss of Protective MeV-Specific Antibody Levels in a Large Proportion of Early-Vaccinated Children

**Figure 1.**
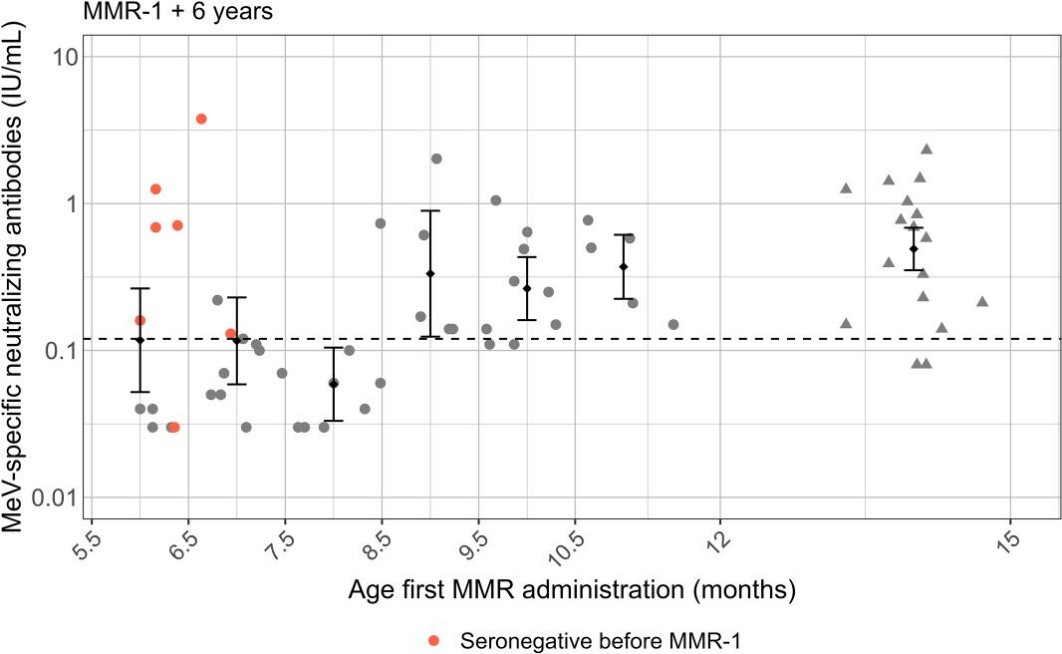
Measles virus–specific neutralizing antibody levels measured 6 years after MMR-1 (at 14 months of age) with the GMC and 95% CIs for the different age groups. The dashed line represents the cutoff for protection against measles (0.12 IU/mL). Early-vaccinated children who had no MeV-specific antibodies detected at 14 months of age are highlighted in orange. Children who did not receive early vaccination are represented by triangles. The LLOQ for the PRNT was established at 0.03 IU/mL. There were 3 values below the LLOQ in the age group 5.5–6.5 months, 1 value for 6.5–7.5 months, and 4 values for 7.5–8.5 months. Abbreviations: GMC, geometric mean concentration; LLOQ, lower limit of quantitation; MeV, measles virus; MMR, measles, mumps and rubella; PRNT, plaque reduction neutralization test.

The MeV-specific neutralizing antibody levels were evaluated 6 years following MMR-1 (Figure 1). The data showed that almost all children who only received 1 dose of MMR at 14 months of age still exhibited antibody levels above the protection cutoff (GMC, .491 IU/mL; 95% CI: .352–.685). The same was found in children who received the additional early vaccination between 8.5 and 12 months of age (GMC [95% CI]: .333 IU/mL [.124–.893], .264 IU/mL [.161–.432), and .371 IU/mL [.225–.613], respectively). However, this was not the case for children who received an additional early MMR vaccination before 8.5 months of age. Five to 6 years after their second MMR dose at 14 months these children had, with a few exceptions, antibody levels below the protective threshold (GMC [95% CI]: .117 IU/mL [.052–.264], .116 IU/mL [.059–.230], and .059 IU/mL [.033–.105], respectively). This shows that MeV-specific neutralizing antibody levels are impacted by the age of first MMR administration (asymptotic general independence test, *P* ≤ .05).

### Early-Vaccinated Children Who Are Seronegative Before MMR-0 Respond Adequately to MMR-1

From the data presented, it was apparent that not all children receiving MMR-0 before 8.5 months of age had lost their MeV-specific neutralizing function, most noticeably in children who received MMR-0 under 7 months of age (Figure 1; orange symbols). Intriguingly, most of these children had no detectable antibodies just before receiving MMR-1 at 14 months and were assumed not to have seroconverted after MMR-0 vaccination (Figure 1 and Supplementary Figure 2, orange symbols). The long-term antibody dynamics of these children were compared with those of children who did not receive early vaccination (Supplementary Figure 3), showing a similar trend over time (between 14 months and 6 years after MMR-1), with no evidence of a difference between these groups (2-sample Brown-Mood median test, *P* = 1). These findings further support the assumption that these children did exhibit an adequate primary immune response to MMR-1.

### Age at First Dose Determines Longevity of Antibodies

**Figure 2.**
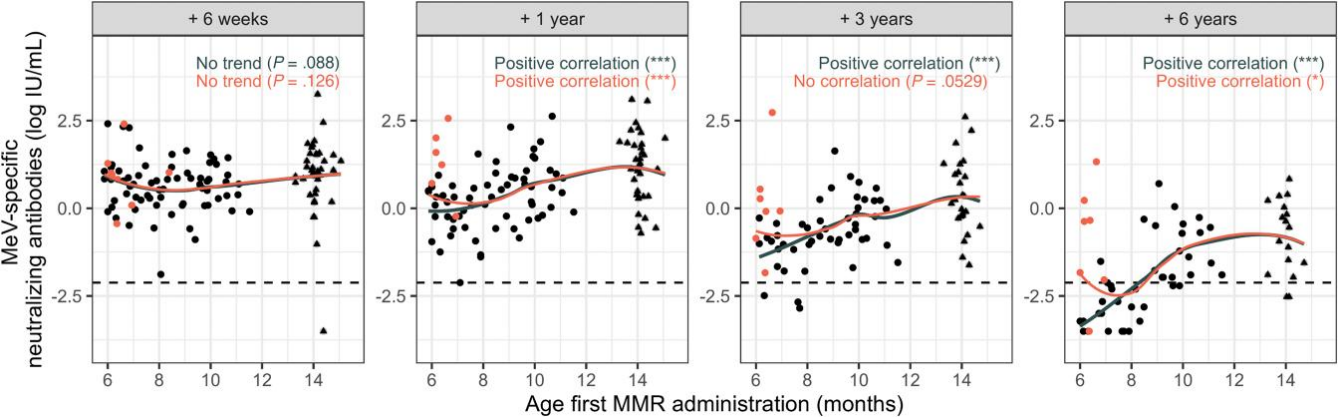
Scatterplots with Loess curves depicting the age-dependent variations in MeV-specific neutralizing antibody levels following MMR vaccination. Each point on the scatterplots represents an antibody level plotted to the age of first MMR vaccination. The dashed line represents the serological protection threshold for measles (0.12 IU/mL). Orange-colored points indicate children who were early-vaccinated but were below the cutoff of protection at 14 months of age (n = 7). Children who did not receive early vaccination are represented by triangles. The Loess curves provide a smoothed representation of the observed trends. The orange Loess curve includes the subgroup of children who were early-vaccinated but were below the cutoff of protection at 14 months of age, while the gray Loess curve excludes these data points. The *P* values indicate the evidence of a correlation between age at first MMR vaccination and MeV-specific neutralizing antibody levels at the given time points as calculated by the asymptotic general independence test. Significance levels: *P ≤ .05, **P ≤ .01, and ***P ≤ .001. Abbreviations: MeV, measles virus; MMR, measles, mumps and rubella.

Next, we examined the MeV-specific neutralizing antibody levels with respect to age at first MMR vaccination at different time points (Figure 2). Six weeks after MMR-1, all children aligned on a single trajectory, where no trend was observed between age at first MMR vaccination and the MeV-specific neutralizing antibody levels. However, an age effect became visible at 1 year post–MMR-1, both when including children who were seronegative before MMR-0 (orange symbols and Loess curve) and when excluding them (gray Loess curve). This age effect became steeper 3 years and 6 years post–MMR-1, associated with accelerated waning in children who had received MMR-0 before 8.5 months of age, being more pronounced when excluding the subset of children who were below the protection threshold at 14 months (orange symbols).

### Early-Vaccinated Children Show Accelerated Antibody Decay

**Figure 3.**
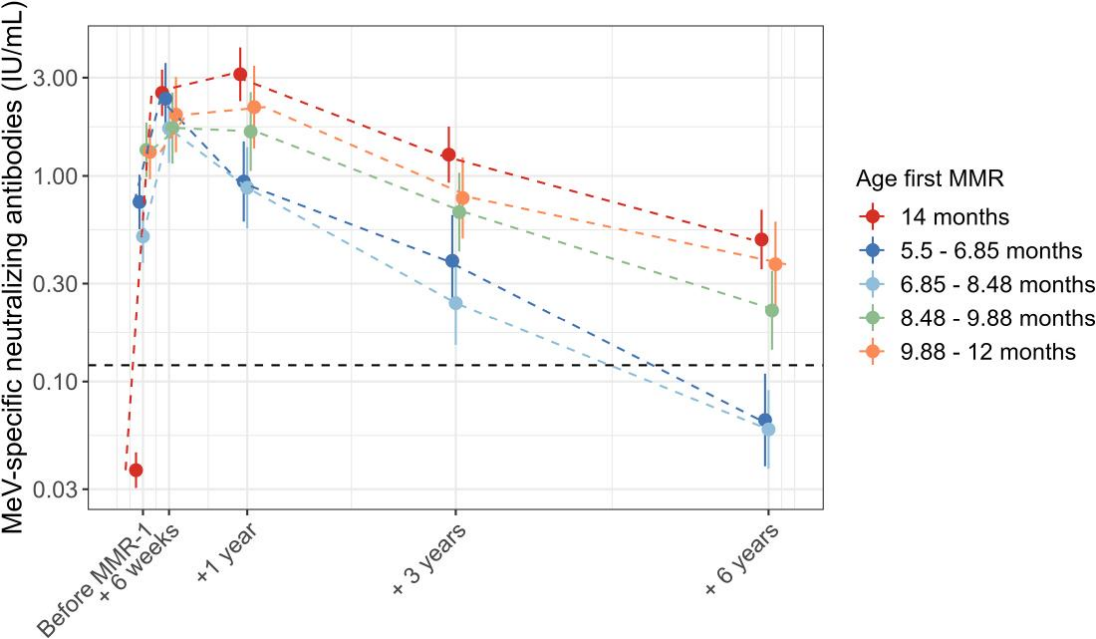
The model-predicted MeV-specific neutralizing antibody level averages shown as error bars with 95% CI of the different age groups. The dashed line represents the cutoff for protection against measles (0.12 IU/mL). In fitting this model, the children who received an early vaccination but were seronegative at 14 months were excluded. Abbreviations: MeV, measles virus; MMR, measles, mumps and rubella.

Due to the individual variability in antibody decay after measles vaccination, we utilized mixed modeling to predict the MeV-specific neutralizing antibody levels over time for the specified age groups (Figure 3). Visual assessment of the residual plots showed less optimal results, while the GMCs calculated with the model were comparable to the observed GMCs, increasing confidence in the model fit (Supplementary Table 1). Since the presented data support the assumption that the children who received MMR-0 but were seronegative at 14 months exhibited an adequate primary immune response to MMR-1, they were excluded from this analysis.

In our modeled antibody dynamics, we observed distinct patterns among the different groups. Children who only received MMR-1 showed an increase in MeV-specific antibody levels from MMR-1 vaccination to 6 weeks, continuing up to 1 year after MMR-1. Those who additionally received MMR-0 between 8.5 and 12 months displayed a similar increase in MeV-specific antibody levels. However, children who received MMR-0 before 8.5 months exhibited waning antibody levels during this period. By 1 year after MMR-1, it became evident that those who received MMR-0 before 8.5 months of age had lower antibody levels compared with those who received early vaccination at later time points or children who did not receive early vaccination.

In addition, we examined the decrease in MeV-specific neutralizing antibody levels between 1 year and 6 years post–MMR-1. Children who received MMR-0 after 8.5 months of age showed a similar decrease as those who were not early-vaccinated (84.2%; 95% CI: 79.4–89%). When MMR-0 was given between 10 and 12 or 8.5 and 10 months, the decrease was 82.7% (95% CI: 75.4–90%) and 86.5% (95% CI: 81.3–91.7%), respectively. However, children who received early vaccination before 8.5 months showed the strongest decrease in MeV-specific neutralizing antibody levels, reaching 92.4% (95% CI: 89.4–95.5%) for children who received MMR-0 between 6 and 8.5 months and 93.2% (95% CI: 90–96.4%) when received between 5.5 and 6.8 months. Most important, in these last 2 groups, this resulted in a decrease below protective antibody levels.

## DISCUSSION

Our research demonstrates that the age at which the first MMR vaccine is administered impacts MeV-specific antibody levels later in life. When MMR-0 was administered before 8.5 months of age, we observed an accelerated decrease in MeV-specific antibody levels over a period of 6 years. Several other studies have reported diminished antibody responses after early measles vaccination, but mainly focusing on short-term outcomes [10, 11, 20]. Our study aimed to track a cohort of children who received an early MMR dose between 6 and 12 months of age over multiple years. Beyond the previously identified lower MeV-specific antibody levels in children vaccinated before 9 months of age, our study reveals that over 70% of children vaccinated before 8.5 months lose their protective antibody levels within 6 years despite having received a repeat MMR dose at 14 months of age.

The observed age-related effect on MeV-specific antibody responses to MMR-0 could result from interference by maternal antibodies or inadequate responses of a developing infant immune system [21, 22]. Existing evidence supports the notion that maternal antibodies can attenuate the immune response to vaccines in infants, although the precise mechanisms remain undetermined [9]. Studies in different animal models, including nonhuman primates, showed that this attenuation could be attributed to viral vaccine neutralization by maternal antibodies, limiting the availability of viral antigens for recognition by the infant immune system [23]. Additionally, mechanisms, such as epitope masking and inhibition of plasma cell and memory B-cell differentiation, have been proposed [24, 25]. Assessing maternal antibody levels was not feasible in our study, and the absence of pre-vaccination samples complicates conclusively ruling out their role in the observed dampened immune response.

The ongoing developing infant immune system in the first year of life could be another explanation for the observed age effect [22]. Early-life, antigen-specific B cells express lower levels of CD80/86 and CD40 coreceptors, limiting their responsiveness and contributing to a reduced antibody production [26]. Notably, early-life bone marrow stromal cells fail to adequately support long-term survival and differentiation of plasmablasts, resulting in the generation of fewer long-lived plasma cells [27, 28]. This phenomenon may explain, besides lower levels of acquired antibodies, a more rapid disappearance of circulating antibodies observed in children vaccinated at an early age, especially for those vaccinated before 8.5 months who showed a more progressive loss of antibodies between 6 weeks and 1 year post–MMR-1, compared with a more enduring response between these time points for those vaccinated after 8.5 months of age.

The MeV-specific humoral immune response is considered T-cell dependent, with the T-cell compartment also undergoing development in infants. The CD4+ T-cell subset in infants exhibits a predominant T-helper 2 (Th2) skewing, emphasizing rapid effector responses with a heavy reliance on innate-like T-cell features [29]. This aims to provide immediate protection during a phase when the capacity for memory formation is still in the developmental stages, compromising long-term protection to early vaccination strategies. Concurrently, the CD8+ T-cell compartment in infants is also undergoing development, as demonstrated by Gans et al [30], who found lower CD8+ T-cell proliferation to measles antigens post-vaccination in infants (6–12 months of age) compared with adults. These findings underscore the intricate interplay of factors in the infant immune system, where a Th2-skewed response, limited memory formation, and impaired B-cell functionality collectively contribute to the diminished immune response observed in children vaccinated with live-attenuated vaccines at an early age. The compromised development of both T- and B-cell compartments highlights the challenges faced in establishing lasting immune protection through vaccination during early infancy.

Among children vaccinated before 8.5 months of age, a subgroup still had protective antibody levels at around 6 years after MMR-1. Most of these children did not seroconvert to MMR-0, likely representing primary vaccine failures. Importantly, these children responded well to MMR-1, a finding consistent with observations in other publications and in routine 2-dose vaccination schedules in which the second dose is given as a second opportunity rather than a booster [8, 10, 12]. Furthermore, supporting the notion of a primary vaccine failure, these children displayed antibody dynamics similar to those who did not receive early vaccination. This underscores the importance of a timely second dose in ensuring protection for infants who encountered primary vaccine failure. Without administration of this subsequent dose, these children would have remained susceptible and without adequate protection.

Importantly, the absence of detectable antibodies does not necessarily mean loss of MeV-specific immunity. Immunity can persist due to immunological memory, allowing the immune system to mount a rapid and effective response upon MeV re-exposure. Therefore, more research including B- and T-cell responses is needed to make definitive conclusions about the potential loss of MeV-specific immunity.

Another key point is that Dutch children receive their second MMR vaccine at 9 years of age, around 8 years after MMR-1. It is possible that this booster improves immunity, albeit that children receiving MMR-0 already received their second MMR vaccine at 14 months of age. Of note, quite a few European countries have implemented a second MMR vaccine around the same time in their regular vaccination program, subsequent to a first MMR vaccine given around 10–12 months of age. Several other studies found evidence that administration of the first MMR vaccine to infants below 9 months of age resulted in lower antibody titers after subsequent doses and decreased duration of immunity compared with when vaccination is started at a later age [11]. This trend of low antibody titers might extend to subsequent generations, because if children receive fewer maternal antibodies due to an increasing number of mothers being vaccinated early, they will become susceptible at an even younger age [5].

In conclusion, the majority of children who received MMR-0 before 8.5 months of age exhibited a decline in antibody levels below the protective cutoff approximately 6 years after MMR-1, despite having received 2 measles vaccinations. This presents a significant concern because, although these children initially developed protective antibody levels during their most vulnerable ages, the diminished responses to MMR revaccinations pose an increased risk for potential future epidemics due to reduced herd immunity. Despite the potential challenges associated with early measles vaccination, it remains an important strategy in specific scenarios where there is a high risk of exposure to MeV, such as during outbreaks or for high-risk populations. In these situations, early vaccination can provide immediate protection against measles, albeit at the expense of a less effective vaccine-induced protection at the later ages.

## Supplementary Material

ciae537_Supplementary_Data

## Supplementary Data

Supplementary materials are available at *Clinical Infectious Diseases* online. Consisting of data provided by the authors to benefit the reader, the posted materials are not copyedited and are the sole responsibility of the authors, so questions or comments should be addressed to the corresponding author.

## Notes

***Author contributions.*** This study was designed by N. Y. R. and R. S. v. B. The laboratory analysis was performed by H. I. t. H. and G. P. S. M. v. d. S. and A. M. N. were responsible for statistical analysis and modeling of the data. The first draft of the manuscript was written by M. v. d. S. and A. M. N., R. L. d. S., J. d. W., N. Y. R., and R. S. v. B. provided input during writing of the manuscript. All authors contributed to the final version of the manuscript. All authors had full access to all the data in the study.

***Data availability.*** Data not publicly available.

***Financial support***. This work was supported by the Dutch Ministry of Health, Welfare, and Sport. The funder of the study had no role in study design, data collection, data analysis, data interpretation, or writing of the report.
