# Supplementary material for: Long-term Dynamics of Measles Virus–Specific Neutralizing Antibodies in Children Vaccinated Before 12 Months of Age
Source: Clin Infect Dis. 2024 Nov 4;80(4):904–10. doi: 10.1093/cid/ciae537 (PMC12043057; doi:10.1093/cid/ciae537)
Supplement: ciae537_Supplementary_Data [file ciae537_supplementary_data.docx]

# Supplemental material


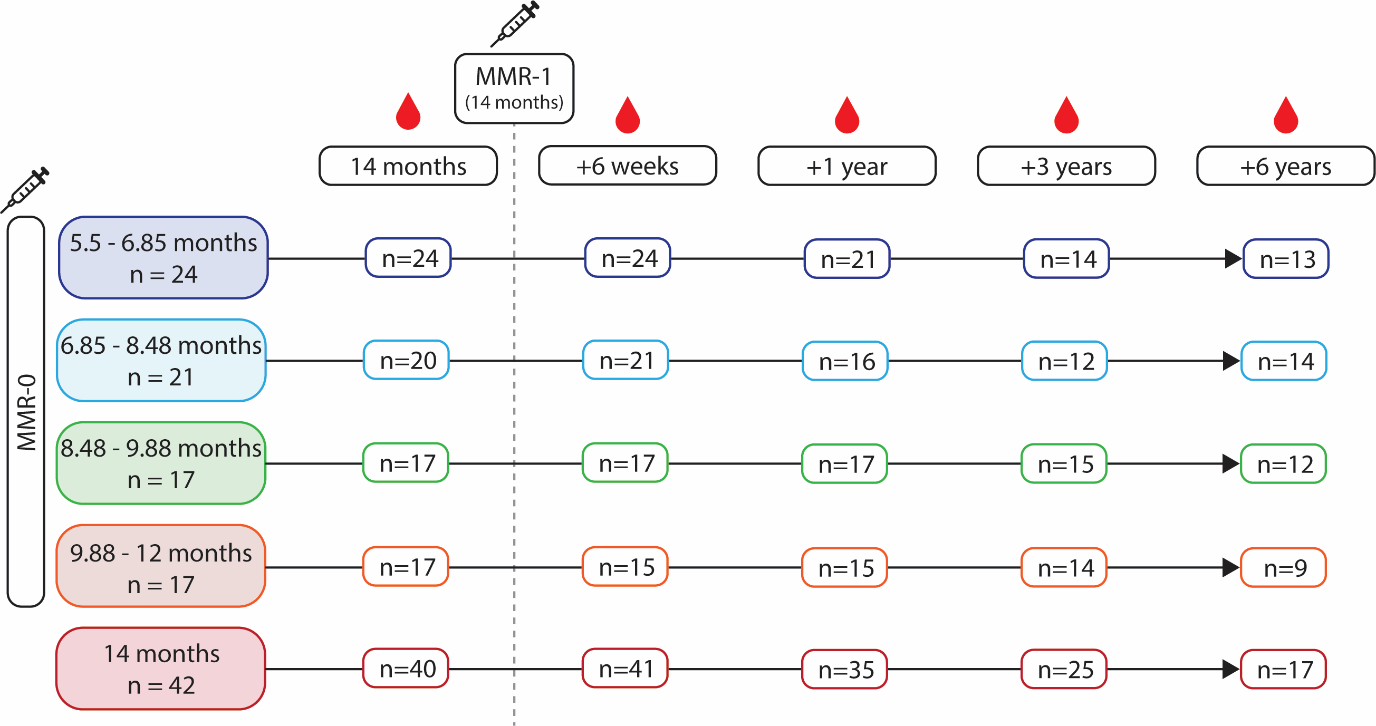


***Supplementary figure 1: Study design.***  *We categorized children based on the age at which they received their first measles, mumps and rubella (MMR) vaccine and indicated the ages at which serum samples were collected. MMR-0 indicated the additional early vaccine between 6 and 12 months of age. MMR-1 denotes the MMR dose administered at 14 months of age.*

***
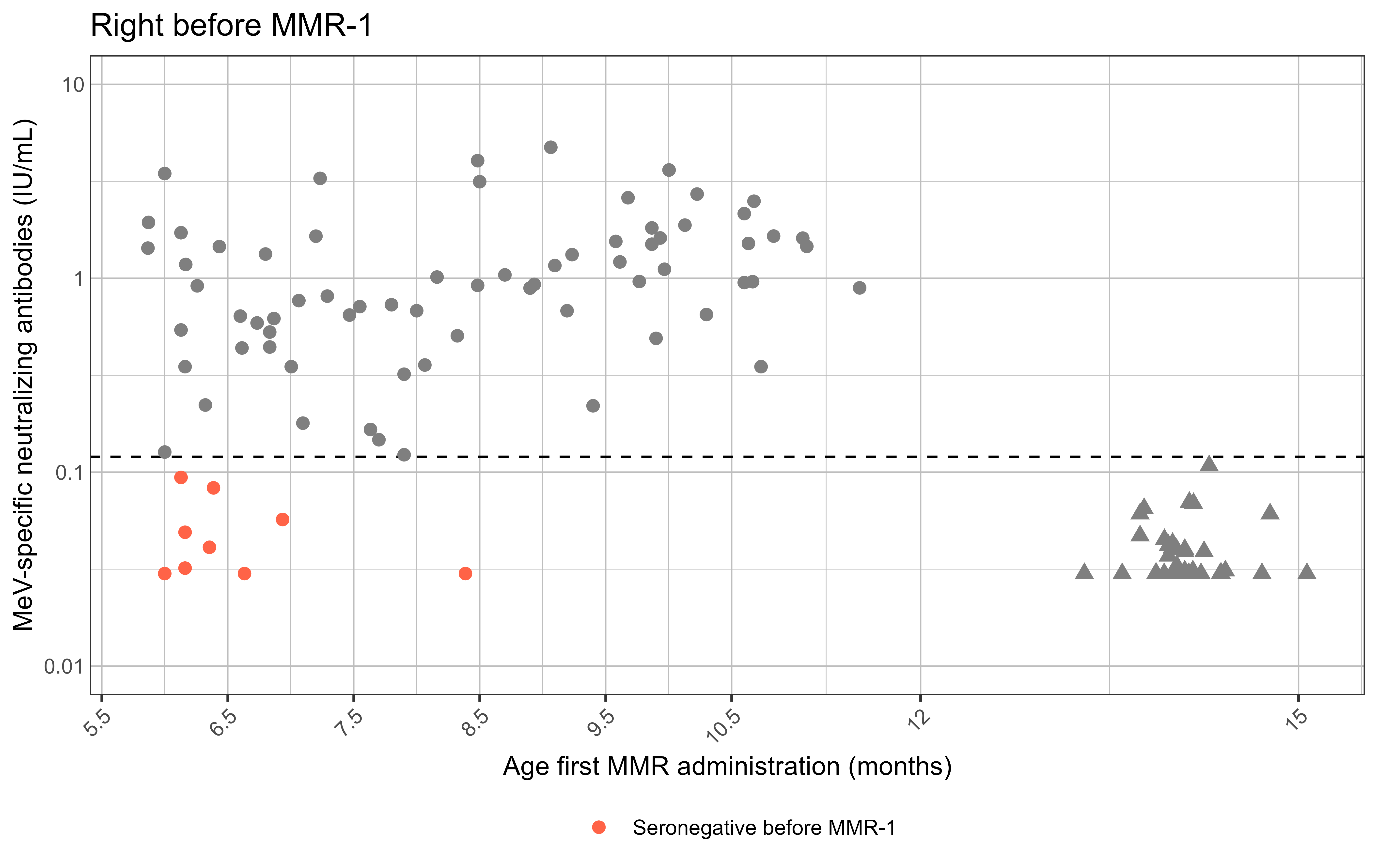
***

***Supplementary figure 2: MeV-specific neutralizing antibody levels measured before MMR-1 at 14 months of age.***  *Children who received MMR-0 before 12 months of age and who did not exhibit protective MeV-specific neutralizing antibody levels (<0*·*12 IU/mL) at this timepoint are highlighted in orange.*

*
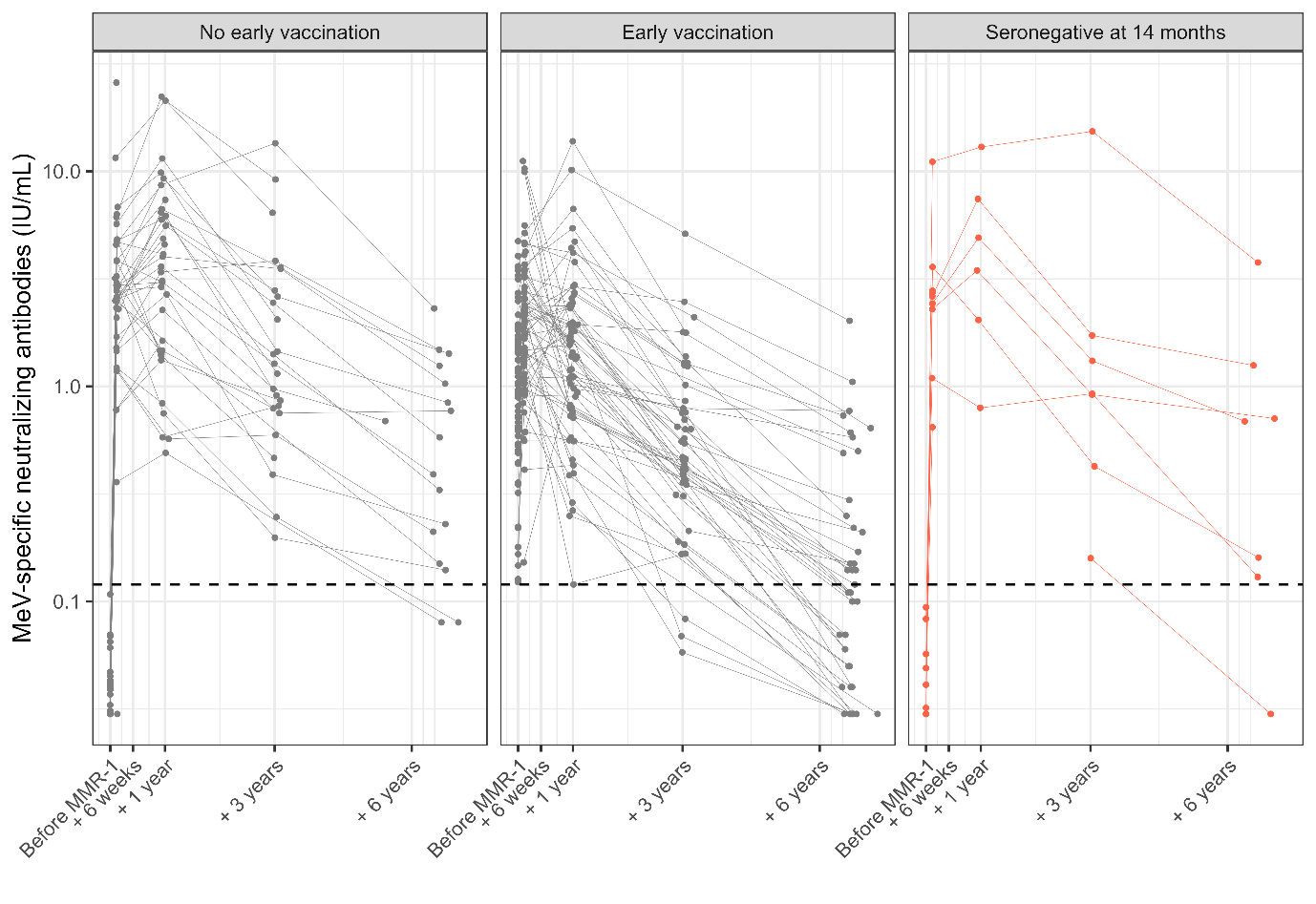
*

***Supplementary figure 3: Individual MeV-specific neutralizing antibody levels over time for children who received no MMR-0 , received MMR-0 between 6 and 12 months of age (early vaccinated) and children that received MMR-0 between 6 and 12 months of age and were seronegative at 14 months of age (seronegative at 14 months).***

***Supplementary table 1: Geometric mean concentration of the MeV-specific neutralizing antibody titer (IU/mL) and corresponding 95%CI for the different age groups at the different timepoints, calculated and predicted with help of the model.*** *In the calculation of these GMCs, the children who had not seroconverted after MMR-0 were excluded.*

| **Age group** | **Before MMR-1** | | **+ 6 weeks** | | **+ 1 year** | | **+ 3 years** | | **+ 6 years** | |
| --- | --- | --- | --- | --- | --- | --- | --- | --- | --- | --- |
|  | **GMC (95%CI) Calculated** | **GMC (95%CI) Predicted** | **GMC (95%CI) Calculated** | **GMC (95%CI) Predicted** | **GMC (95%CI) Calculated** | **GMC (95%CI) Predicted** | **GMC (95%CI) Calculated** | **GMC (95%CI) Predicted** | **GMC (95%CI) Calculated** | **GMC (95%CI) Predicted** |
| **[5·5, 6·85]** | 0·747  (0·490-1·140) | 0·747  (0·551-1·013) | 2·373  (1·532-3·674) | 2·373  (1·593-3·534) | 0·986  (0·711-1·366) | 0·938  (0·586-1·502) | 0·349 (0·242-0·504) | 0·381  (0·227-0·639) | 0·050 (0·035-0·071) | 0·064  (0·38-0·108) |
| **(6·85, 8·48]** | 0·509  (0·337-0·769) | 0·509  (0·379-0·684) | 1·500  (1·062-2·120) | 1·704  (1·157-2·509) | 0·654 (0·291-1·467) | 0·777  (0·481-1·255) | 0·245 (0·168-0·359) | 0·247  (0·154-0·398) | 0·055 (0·042-0·072) | 0·059  (0·038-0·091) |
| **(8·48, 9·88]** | 1·338  (0·930-1·923) | 1·338  (0·987-1·814) | 1·708  (1·211-2·410) | 1·708  (1·147-2·544) | 1·643  (1·096-2·461) | 1·643  (1·034-2·609) | 0·720 (0·466-1·113) | 0·667  (0·429-1·039) | 0·269 (0·149-0·454) | 0·222  (0·143-0·345) |
| **(9·88, 12]** | 1·307  (0·961-1·779) | 1·307  (0·964-1·772) | 2·095  (1·568-2·801) | 1·985  (1·304-3·022) | 2·323 (1·487-3·629) | 2·147  (1·321-3·490) | 0·842 (0·597-1·188) | 0·783  (0·497-1·234) | 0·352 (0·253-0·489) | 0·372  (0·231-0·598) |
| **(12, 15.5]** | 0·028  (0·023-0·035) | 0.037 (0·030-0·045) | 2·528  (1·821-3·512) | 2·533 (1·953-3·283) | 3·220 (2·360-4·395) | 3·116  (2·272-4·273) | 1·434 (1·025-2·005) | 1·269  (0·925-1·743) | 0·452 (0·324-0·630) | 0·492  (0·352-0·686) |

*_Abbreviation: MMR-1: Measles, Mumps and Rubella vaccine at 14 months of age_*
